# Following the Mechanisms of Bacteriostatic *versus* Bactericidal Action Using Raman Spectroscopy

**DOI:** 10.3390/molecules181113188

**Published:** 2013-10-24

**Authors:** Silvie Bernatová, Ota Samek, Zdeněk Pilát, Mojmír Šerý, Jan Ježek, Petr Jákl, Martin Šiler, Vladislav Krzyžánek, Pavel Zemánek, Veronika Holá, Milada Dvořáčková, Filip Růžička

**Affiliations:** 1 Institute of Scientific Instruments of the Academy of Science of the Czech republic, v.v.i., Královopolská 147, 612 64 Brno, Czech Republic; E-Mails: berns@isibrno.cz (S.B.); pilat@isibrno.cz (Z.P.); sery@isibrno.cz (M.Š.); jezek@isibrno.cz (J.J.); jakl@isibrno.cz (P.J.); siler@isibrno.cz (M.Š.); vlk@isibrno.cz (V.K.); pavlik@isibrno.cz (P.Z.); 2 Department of Microbiology, Faculty of Medicine and St. Anne’s Faculty Hospital, Brno, Czech Republic; E-Mails: veronika.hola@fnusa.cz (V.H.); milada.dvorackova@fnusa.cz (M.D.); fruzic@fnusa.cz (F.R.)

**Keywords:** Raman spectroscopy, antibiotics, bacteria, bactericidal, bacteriostatic

## Abstract

Antibiotics cure infections by influencing bacterial growth or viability. Antibiotics can be divided to two groups on the basis of their effect on microbial cells through two main mechanisms, which are either bactericidal or bacteriostatic. Bactericidal antibiotics kill the bacteria and bacteriostatic antibiotics suppress the growth of bacteria (keep them in the stationary phase of growth). One of many factors to predict a favorable clinical outcome of the potential action of antimicrobial chemicals may be provided using *in vitro* bactericidal/bacteriostatic data (e.g., minimum inhibitory concentrations—MICs). Consequently, MICs are used in clinical situations mainly to confirm resistance, and to determine the *in vitro* activities of new antimicrobials. We report on the combination of data obtained from MICs with information on microorganisms’ “fingerprint” (e.g., DNA/RNA, and proteins) provided by Raman spectroscopy. Thus, we could follow mechanisms of the bacteriostatic *versus* bactericidal action simply by detecting the Raman bands corresponding to DNA. The Raman spectra of *Staphylococcus epidermidis* treated with clindamycin (a bacteriostatic agent) indeed show little effect on DNA which is in contrast with the action of ciprofloxacin (a bactericidal agent), where the Raman spectra show a decrease in strength of the signal assigned to DNA, suggesting DNA fragmentation.

## 1. Introduction

**Figure 1:**
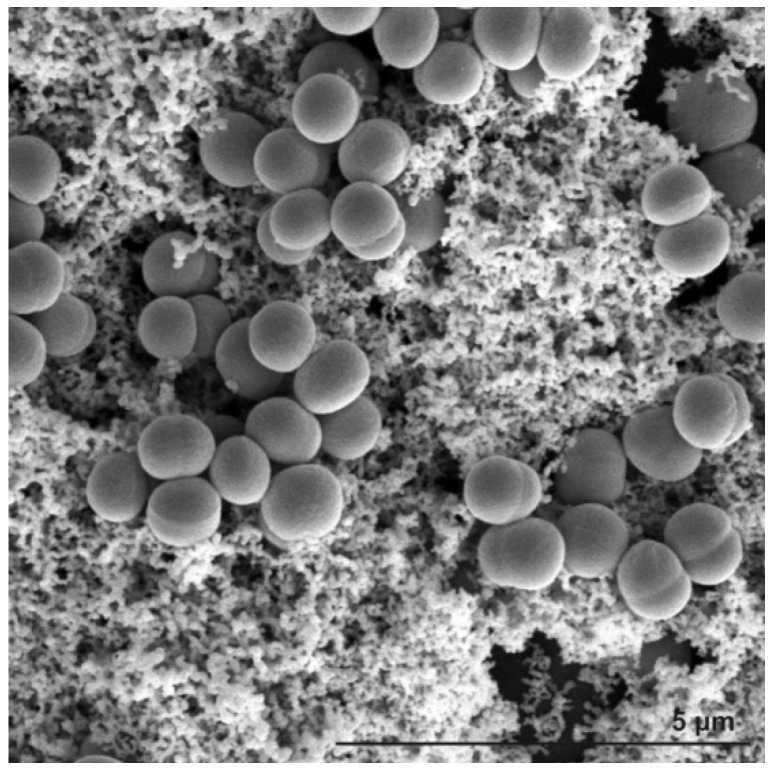
SEM image of *Staphylococcus epidermidis* grown on a glass substrate. Biofilm (slime) formation is clearly visible throughout the sample filling the space between grape-like clusters of *Staphylococcus* colonies.

The clinical microbiology laboratory often faces a typical problem which is to distinguish between contaminant and invasive isolates [1,2,3]. Moreover, interpretation of the clinical relevance of each isolate by the fast detection of the ability to form biofilms (which is an important virulence factor) should be provided. For example, biofilm-positive *S. epidermidis* isolates can be considered as more virulent and invasive (see Figure 1) when compared to the *S. epidermidis* isolates that do not form a biofilm (biofilm-negative) and can be considered as contaminants. Consequently, the main task is the prediction of *in vitro* antibiotic susceptibility testing for prognosis of the clinical response to treatment and for guidance on the selection of proper antibiotic against invasive isolates resulting in a need for a rapid assessment of the clinical response of considered antibiotics. Therefore, the availability of such a rapid technique would be of great advantage for choosing an appropriate therapeutics strategy.

Raman spectroscopy has been presented in many studies as a technique that provides rapid identification and discrimination of medically relevant microorganisms, bacteria, and biological samples based on its ability to detect and identify important molecular complexes in biological samples [4,5,6,7,8,9,10,11,12,13,14,15]. Extensive effort of the Raman Research Group at Gent University has resulted in the first database of Raman features of biological samples [16].

Our investigation presented in this paper expands our earlier analysis of bacterial strains, including a series of *S. epidermidis* clones [1,9], using Raman spectroscopy and shows that this method is able to identify important molecules in biological samples.

Fluoroquinolones and β-lactam antibiotics are examples of bactericidal antibiotics that completely eradicate the infectious agent. Fluoroqinolones influence DNA metabolism by inhibition of the DNA gyrase (topoisomerase II) and this leads to DNA fragmentation [17]. β-Lactam antibiotics, including penicillins, inhibit bacterial cell wall synthesis. In contrast, clindamicin and chloramphenicol are examples of bacteriostatic antibiotics that slow or stop the bacterial growth, usually by the inhibition of protein synthesis. As a result, the infectious agent is then much more easily eliminated by the immune system [18]. The distinction between bactericidal and bacteriostatic agents appears to be clear according to the *in vitro* definition, but this only applies under strict laboratory conditions and is inconsistent for a particular agent against all bacteria. In reality there are not two pure categories of antimicrobial agents. Most antibacterials are better described as potentially being both bactericidal and bacteriostatic [18]. We like to note here that although all quinolones are bactericidal, they have a single concentration at which they are most bactericidal: the paradoxical effect of decreased killing at higher concentration most likely results from dose-dependent inhibition of RNA synthesis [18]. In order to determine the susceptivity of organisms to antibiotics we used MIC (minimum inhibitory concentration) plate values [19,20,21].

Due to possible modification of the bacterial DNA during the treatment we monitored the effect of both groups of antibiotics (bactericidal and bacteriostatic) on the Raman peak corresponding to phosphorus diester bonds (O-P-O, 785 cm^−1^) concentrated in DNA [1,11,12]. In order to standardize peak heights of DNA from the selected spectral region we opted, after numerous tests, to use the peak intensity of phenylalanine. It should be noted that antibiotics may well inhibit protein synthesis so that concentration of amino acids in the cells might be reduced. However, in order to assure that our measured data can be quantified we checked the magnitude of phenylalanine peak which decreased to approximately 90% of the initial value. For the DNA a steep decrease was observed down to 5%–10% of the initial value. While not ideal, this procedure could be an alternative in instances where no proper standards are available.

The main aim of our investigations is to focus on the fact that Raman spectroscopy can potentially provide an insight into the mechanism of antibacterial agents at the single cell level. We further demonstrate the potential of Raman spectroscopy as a technique which can explore biomolecular responses in selected bacteria.

## 2. Results and Discussion

**Figure 2:**
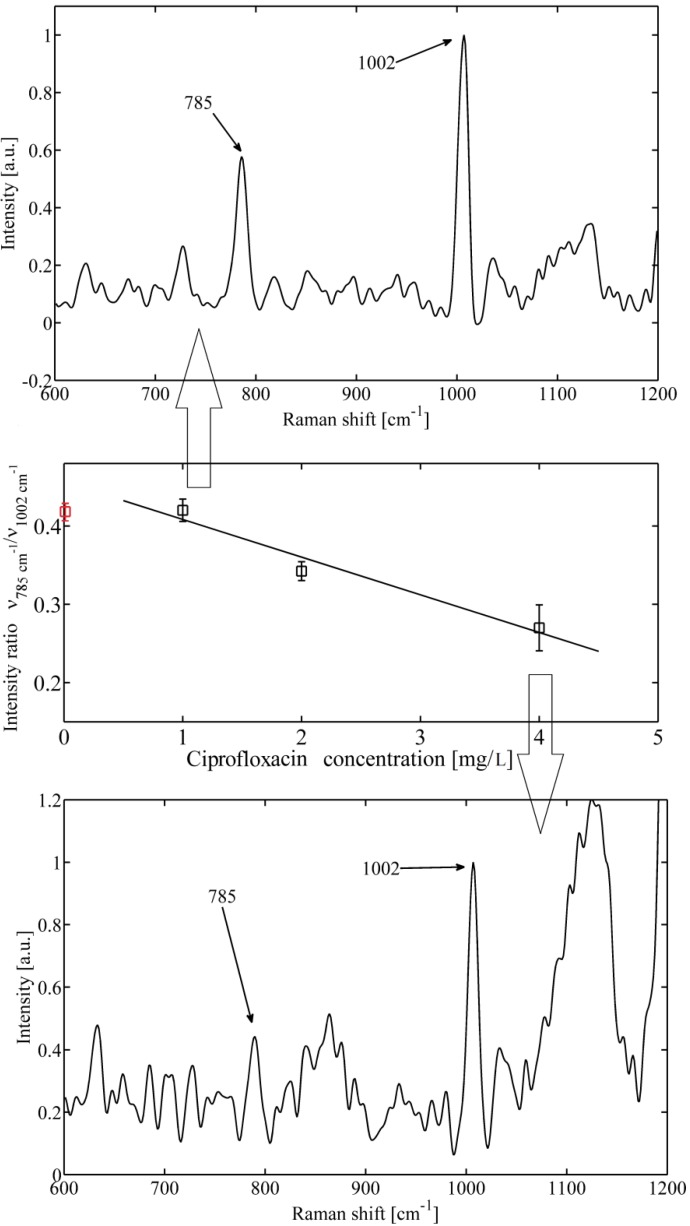
An example of *Staphylococcus epidermidis* response to bactericidal action of ciprofloxacin. (**a**) Top—Raman spectrum obtained for low antibiotic concentration (1 mg/L). The DNA peak at 785 cm^−1^ is clearly visible together with phenylalanine peak at 1002 cm^−1^, (**b**) Middle—mean value of ratio of maximum of Raman intensity at 785 cm^−1^* versus* 1002 cm^−1^ for different concentration of applied ciprofloxacin measured from 10 different cells. Mean value of a control sample not exposed to antibiotic (red circle) is shown. (**c**) Bottom—Raman spectrum obtained for higher antibiotic concentration (4 mg/L). The DNA peak at 785 cm^−1^ disappeared which indicates fragmentation of DNA. Note that the two peaks shown here (around 1130 cm^-1^) could not be reliably determined from the analysis of the data.

The most prominent features observed in the Raman spectra of bacteria can be broadly categorized into four groups–proteins, DNA/RNA, sugars, and lipids (for details see, e.g., [1]). In our case DNA contributes to the characteristic peak at 785 cm^−1^ formed by vibration of phosphorus diester (O-P-O) bonds [11,12]. When examining the peak at 785 cm^−1^ for cells exposed to the bactericidal substance ciprofloxacin (Figure 2a,c), a distinct difference in their intensity and shape can be observed. A decrease in the band intensity at higher antibiotic concentration, shown in Figure 2c, suggests a lesser number of phosphorus diester (O-P-O) bonds, thus showing a greater fragmentation of DNA leading to death of *S. epidermidis* cells. The explanation is coherent with the mechanism of how fluoroqinolon influences the metabolism of DNA, as it was explained above. 

**Figure 3:**
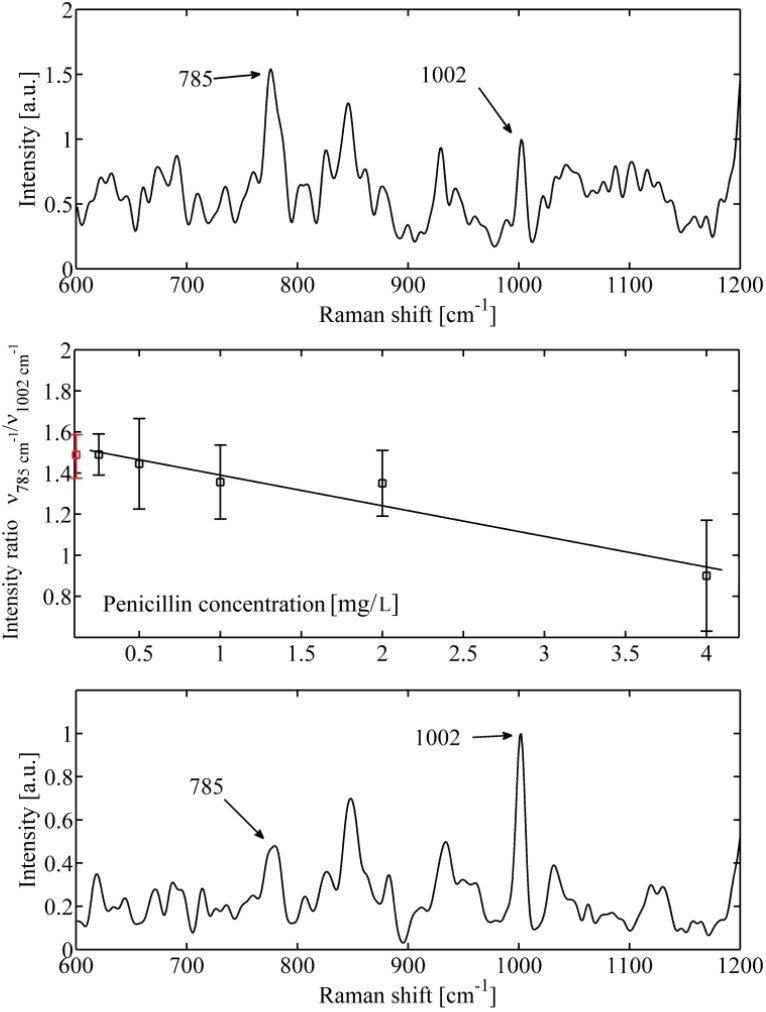
An example of *Staphylococcus epidermidis* response to bactericidal action of penicillin. (**a**) Top—Raman spectrum obtained for low antibiotic concentration (0.2 mg/L). The DNA peak at 785 cm^−1^ is clearly visible together with phenylalanine peak at 1,002 cm^−1^. (**b**) Middle—mean value of ratio of maximum of Raman intensity at 785 cm^−1^* versus* 1,002 cm^−1^ for different concentrations of applied penicillin measured from 10 different cells. Mean value of a control sample not exposed to antibiotic (red circle) is shown. (**c**) Bottom—Raman spectrum obtained for higher antibiotic concentration (4 mg/L). The DNA peak at 785 cm^−1^ nearly disappeared which, again, indicates damage of DNA.

Cells exposed to the bactericidal substance penicillin confirmed the same behaviour of DNA peak as those exposed to ciprofloxacin (Figure 3). However, one would not expect penicillin, a drug known to inhibit cell wall formation, to show this relationship. This could be explained by the fact that all major classes of bactericidal antibiotics including aminoglycosides, quinolones, and β-lactams, promote the generation of lethal hydroxyl radicals in bacteria, despite the stark differences in their primary drug-target interactions [22]. Consequently, hydroxyl radical generation (through the Fenton reaction) contributes to the killing efficiency of these lethal drugs resulting in bacterial cell death [22,23]. Hydroxyl radicals are extremely toxic and readily damage proteins, membrane lipids, and DNA. Thus, the reduction of DNA could be due to cell death combined with mechanisms leading to DNA fragmentation.

As was mentioned, one of the markers for the presence of proteins can be found at 1002 cm*^−^*^1^ and is contributed by phenylalanine vibrations [11,12]. Thus, we normalized the DNA peak intensity to phenylalanine for each spectrum. If the mean value of this ratio varies with the concentration of antibiotics (Figure 2b and Figure 3b), it suggests that the DNA is fractionated and the antibiotic actually kill the organisms.

**Figure 4:**
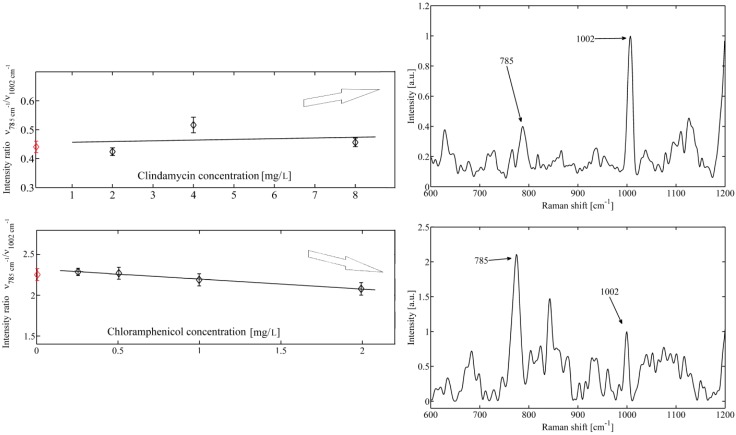
Mean value of bacteria response to bacteriostatic action of (**a**) top–clindamycine and (**b**) bottom–chloramphenicol, on cells of *Staphylococcus epidermidis*. Constant intensity ratio indicates for both antibiotics no visible influence on bacterial DNA. Raman spectra for control samples are shown on the right (indicated with red circles).

In contrast, if the mean values of the normalized DNA peak do not change significantly with the concentration of the antibiotic then the concentration of the related DNA bonds is not changed in the investigated bacteria. Figure 4 shows an example of DNA band when *S. epidermidis* cells are exposed to bacteriostatic clindamycine and chloramphenicol. It is seen that a nearly flat line is observed, suggesting that the intensity of the DNA peak does not change with the concentrations of drug. This finding is not surprising because bacteriostatic clindamycine and chloramphenicol should not affect metabolism of nucleic acid and cells should survive relatively undamaged and viable. Also, bacteriostatic antibiotics, do not induce the production of hydroxyl radicals which contribute to the killing efficiency of antibiotics.

## 3. Experimental

### 3.1. Experimental Setup and Procedures

**Figure 5:**
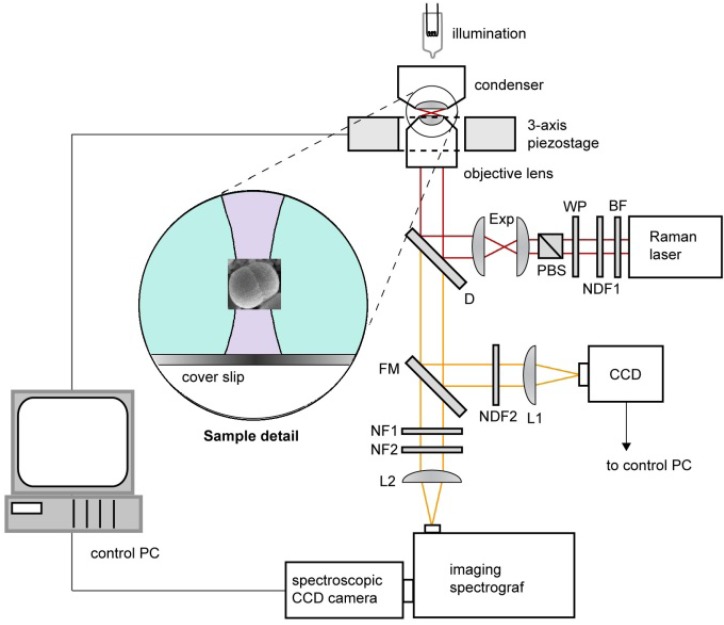
Schematic diagram of the Raman tweezers setup where the same laser beam is used for optical trapping and Raman scattering. BF–band pass filter, D–dichroic mirror, Exp–beam expander, FM–flipping mirror, L1,2–lenses, NDF1,2–neutral density filters, NF1,2–notch filters, PBS–polarizing beam splitter, WP–lambda-half wave plate. Inset shows the detail of optically trapped bacterium near the focus the laser beam at the wavelength 785 nm.

Experiments were carried out using a custom-built experimental system of Raman tweezers (see Figure 5). It combines the Raman microspectrometer with optical tweezers [24,25,26] providing spatial confinement of individual bacterial cells during the Raman spectrum acquisition. The same laser beam is used for optical trapping and Raman spectroscopy. In order to minimize the background fluorescence from the studied cell as well as the potential photo damage of the trapped cell, we chose laser wavelength close to the near-infrared spectral region. The output beam from a laser (output power ~1W, λ = 785 nm, beam diameter 0.6 mm Ti: Sapphire, Coherent 899/01, Santa Clara, CA, USA) was delivered to the setup by an optical fibre and its diameter was expanded 3× by an external telescope (not shown in Figure 5). Behind the telescope the beam passed through a bandpass filter BF (transmission bandwidth 3 nm centred on 785 nm; MaxLine LL01-785, Semrock, Rochester, NY, USA) in order to get rid of parasitic laser wavelengths. The power of the Raman laser beam was roughly adjusted by a neutral density filter NDF1 and fine setting was done by a combination of a λ/2 wave plate WP and a polarizing beam splitter PBS. Beam diameter was further enlarged 2× by beam expander Exp. The laser beam was further coupled to the microscope frame via a dichroic mirror D (LPD01-785RS, Semrock) and focused on the specimen with a water-immersion objective lens (UPLSAPO 60×, NA 1.20, Olympus, Tokyo, Japan). Water immersion objective provides 3D optical trapping of bacteria even several tens of micrometers deep in the sample without significant decrease of the optical trap performance [27]. The maximal available laser power at the specimen plane was approximately 150 mW. The objective was mounted on a custom-made aluminium frame that also provided a stable support for sample illumination path and 3-axis piezo-driven stage (P-517.3CL, Physik Instrumente, Karlsruhe, Germany) positioning the sample relative to the beam focus.

About 50 µL of sedimented bacterial culture treated with antibiotic were taken from a MIC plate, placed on the top of a coverslip and dispersed in about 100 µL of water filtered through 0.22 µm pores. The cells were observed by a standard CCD camera through the flipping mirror FM. The individual microorganism was optically confined at the laser beam focus placed approximately 20 μm above the liquid-glass interface. During the acquisition of the Raman spectrum (which lasted, varied depending on the sample, from 10 s to 100 s) the flipping mirror FM was flipped down and the sample illumination was switched off. The Raman scattered light from the trapped microorganism was collected by the same water-immersion objective, focused by a lens L2 on the entrance slit of an imaging spectrograph (focal length 300 mm, f/3.9, 600 gr/mm diffraction grating, SpectraPro 2300i, PI Acton, Acton, MA, USA), imaged on the chip of a high-sensitivity liquid-nitrogen-cooled spectroscopic CCD camera (Spec-10:100BR/LN, Princeton Instruments, Acton, MA, USA), and recorded using the camera control software (WinSpec, Acton, MA, USA). Recorded spectra were processed off-line using custom-written routines implemented in Matlab software (MathWorks, Natick, MA, USA). Much stronger Rayleigh scattered light at the laser wavelength was blocked by two edge filters NF1 (ZX000626, Iridian, Ottawa, Canada) and NF2 (LP02-785RS, Semrock) and did not enter the spectrograph.

For our Raman instrument we have estimated that the full axial extent (depth) _z of the excitation region was measured to be approximately 4 μm. This value is comparable with the diffraction limit expected for focusing λ = 785 nm light with an NA = 1.2 microscope objective in water. Thus, we can expect the full lateral extent (width) of the excitation region also attains the diffraction-limited value Δx = 1.22λ/NA ~ 0.8 μm. Considering that the bacterial cells are less then 800 nm in diameter, we assume that only a few cells (from 1 to about 5) are trapped and analyzed in the trapping region of 0.8 µm. The cells are exposed to the laser light only when the data are acquired–typically 10–20 s. After the exposure the laser is blocked so that photo-bleaching of the cell is minimal. Consequently, laser waits for new cells to be trapped/tested for another data set. Collecting Raman signal from only a few cells results in noisy spectra which make difficult to estimate further changes in the Raman spectra except for selected two strong peaks. We would like to note, that our results warrant more extensive investigations with larger collections of clinical *S. epidermidis* strains to fully evaluate the discriminatory power of Raman spectroscopy.

### 3.2. Spectrum Processing and Analysis

In order to extract quantitative information from the acquired Raman spectra, we adopted the high-pass signal filter (Rolling Circle Filter–RCF [28]) to separate narrow Raman spectral peaks from the wide spectral background. With an appropriate choice of the filter parameters (filter width and number of filter passes); background can be effectively removed without causing a significant distortion of the signal peaks. We kept the same filter parameters for all the measurements presented in this paper. As an example–Figure 2a,c show a typical background-corrected Raman spectrum obtained from a single *Staphylococcus epidermidis* cell. The most prominent Raman spectral peaks correspond to DNA (785 cm^−1^) and phenylalanine (1002 cm^−1^).

### 3.3. Sample Preparation

For *in vivo* Raman microspectroscopic experiments the exposure of tested *staphylococci* to the different concentration of selected antibiotic was carried out using the standard MIC testing by broth microdilution methods according to the Clinical and Laboratory Standards Institute (CLSI) protocol [29]. *S. epidermidis* CCM 4418 (Czech Collection of Microorganisms, Brno, Czech Republic) was selected as a valid candidate for our study. The following serial dilutions of selected antibiotics were used: ciprofloxacin hydrochloride (0.063 to 8 μg/mL), clindamicin hydrochloride (0.063 to 8 μg/mL), chloramphenicol (0.25 to 32 μg/mL), and penicillin G potassium salt (0.031 to 4 μg/mL). All antibiotics were from Discovery Fine Chemicals (Wimborne, United Kingdom). Each concentration of particular antibiotic was inoculated by tested strain according to CLSI protocol. After incubation at 35 °C for 24 h the reading and interpretation of the MIC was performed. MIC is defined as the lowest concentration of antibiotic at which there is no visible growth of the organism after incubation [18,20]. Out of this selection ciprofloxacin and penicillin belong to the bactericidal antibiotic. Clindamicin and chloramphenicol are bacteriostatic antibiotics. We would like to note that clindamicin and chloramphenicol could be considered as possible/supplementary candidates for treatment of MRSA (methicillin-resistant *Staphylococcus aureus*) [19]. We sampled bacterial suspension from each concentration of tested antibiotics in wells where visible bacteria growth could be seen, e.g., for ciprofloxacin we could use only three concentrations before MIC was reached. Thus, depending on the drug [21], we have obtained different numbers of sampling points (Raman spectra) for selected antibiotic. As it was mentioned above a drop of bacterial culture was placed directly on a microscope coverslip/microfluidic chip. Care was taken to introduce the sampling volume immediately on the plate to avoid unnecessary stress on the cells. Consequently, the cells were analyzed with Raman microspectroscopy.

## 4. Conclusions

We have demonstrated that Raman spectroscopy is able to distinguish individual bacteria treated with bactericidal or bacteriostatic antibiotics. In the case of bactericidal antibiotics we have observed decrease of intensity of DNA Raman peak with respect to the phenylalanine. This suggests that the bactericidal antibiotic has indeed a visible effect on bacterial DNA during incubation in MIC. On the contrary, bacteriostatic antibiotic does not visibly influence DNA, so that significant decrease of DNA feature was not observed. To the best to our knowledge this is the first result where Raman spectroscopy can follow the mechanism of antibacterial chemicals at the single cell level. Selected antibiotics, namely ciprofloxacin, penicillin, and clindamycin, chloramphenicol were used in this feasibility study as representatives of bactericidal and bacteriostatic antibiotics, respectively. This is encouraging result because it proves Raman spectroscopy as a tool for monitoring biological changes introduced by antibiotics. Thus, the potential benefits of our investigation for examining the mechanisms of novel antibiotics have been shown. At present, we continue our efforts to prove the discriminatory power of Raman spectroscopy for clinical diagnostics. Specifically, for this to succeed systematic studies are still required to investigate reaction of bacteria on different antibiotic treatment.
